# Clinical education programs for new graduate nurses: a scoping review

**DOI:** 10.1186/s12912-026-04561-8

**Published:** 2026-03-24

**Authors:** Jennifer McClure, Catherine Gonzalez, Hind Elmir, Shanisha Prasad, Nalini Singh, Leanne Senior, Josephine S. F. Chow, Gladis Kabil, Sheeja Perumbil Pathrose

**Affiliations:** 1 Liverpool Hospital, South Western Sydney Local Health District, Sydney, Australia; 2 Education and Organisational Development Service, South Western Sydney Local Health District, Sydney, Australia; 3 SWS Nursing and Midwifery Research Alliance, South Western Sydney Local Health District, Sydney, Australia; 4 Ingham Institute for Applied Medical Research, Sydney, Australia; 5 Faculty of Medicine, University of New South Wales, Sydney, Australia; 6 NICM Health Research Institute, Western Sydney University, Sydney, Australia; 7 University of Tasmania, Hobart, Australia; 8 School of Nursing and Midwifery, Western Sydney University, Sydney, Australia; 9 JBI Western Sydney Centre, University of Adelaide, Adelaide, Australia; 10 Westmead Hospital, Western Sydney Local Health District, Sydney, Australia

**Keywords:** Clinical education, Competence, Confidence, Job satisfaction, New graduate nurses, Retention, Scoping review, Transition to practice, Residency programs

## Abstract

**Objective:**

To explore the clinical education programs that support new graduate nurses and their impact on confidence, competence, job satisfaction and workforce retention.

**Background:**

Nursing workforce turnover is a key issue for healthcare organisations, impacting efficiency, cost-effectiveness, quality of care and patient safety. New graduate nurses are an at-risk cohort who face distinct challenges transitioning into practice, requiring structured and supportive clinical education programs.

**Methods:**

The JBI methodology for scoping review approach was used. The review was registered and made publicly available in Open Science Framework: https://osf.io/ke5hd. Included studies involved clinical education programs that support new graduate nurses within their first 12 months of employment after completing a bachelor-level degree and their impact on confidence, competence, job satisfaction and retention. The literature search was conducted in March 2025 using CINAHL, Medline and Scopus, with no restrictions on geography or language, and included grey literature across the selected databases.

**Results:**

31 studies met the inclusion criteria of the review, with the majority (*n* = 27) conducted in a hospital setting. Various terminologies were adopted for clinical education programs in the included studies, such as transition or residency programs, preceptorship programs and critical reflection programs. The duration of these programs was between six weeks to 18 months, with the majority delivered over 12 months (*n* = 18). Clinical education modalities included education sessions, support strategies, facility orientation, leadership involvement in structured programs, shift pattern rules, reflection, structured feedback and participation in quality improvement projects. This review highlighted the positive impact of providing clinical and social support to this at-risk cohort, and identified that structured clinical education programs resulted in improved confidence, competence, retention and job satisfaction of new graduate nurses. Additionally, the review of these studies revealed organisational challenges, including staffing issues and competing demands between sustaining clinical services, and releasing new graduate nurses for scheduled educational activities required to meet clinical education objectives.

**Conclusion:**

The review identified that structured clinical education programs positively impact the transition of new graduate nurses into professional practice. These findings will inform healthcare educators, policymakers and leaders in planning and prioritising responsive strategies to strengthen new graduate clinical education programs and sustain a future-ready nursing workforce.

**Supplementary Information:**

The online version contains supplementary material available at 10.1186/s12912-026-04561-8.

## Background

Nurses play a critical role in delivering safe, effective and person-centred care [1] across all settings, from acute hospitals to primary care and community-based services [2]. Globally, the nursing workforce is ageing, with fewer young nurses entering the profession, juxtaposed with the baby boomer generation, who are on the verge of retirement [3]. The World Health Organization warns of the challenges to nursing education, employment, retention and performance due to the demand for a competent and well-prepared nursing workforce globally [4]. The issue of an ageing workforce has added to the pressure and demands on healthcare systems [5]. Another contributing factor to attrition is that nurses are leaving the profession due to multiple challenges, including physical and emotional stress, insufficient resources and a heavy workload [6]. Given this, workforce sustainability is a global challenge and new graduate nurses (NGNs) are often central to strategic workforce models [7].

NGNs entering professional practice must quickly adjust from a structured classroom setting to a fast-paced, high intensity clinical environment in which they must think critically [8]. The gap between theoretical learning and practical work is not specific to nursing and has been described as a factor for many professions that may historically have had an apprenticeship training model [9]. The transition from student to professional nurse is an important milestone, yet it is marked with multifaceted struggles relating to application of theoretical knowledge in practical settings, navigating an unfamiliar environment and assimilating to workplace culture [8]. In addition, NGNs often leave due to multiple factors such as inadequate supervisor and peer support, challenges in the workplace and work environments [10]. Retaining NGNs is critical not only to mitigate the financial burden of turnover [11], but to sustain a healthy nursing workforce and encourage long-term workforce retention.

To support the successful integration of NGNs into the complexity of the healthcare system, effective clinical education programs must be adopted. Transition strategies are often formalised programs provided by employers in the first year of practice following completion of a nursing degree [12], while standalone educational strategies are used to facilitate specific learning needs. These approaches are shaped by workplace culture and resource availability and are influenced by the quality and consistency of the clinical learning environment [13]. Structured clinical education programs have emerged to facilitate transition to practice for NGNs [14], and involve a combination of approaches such as preceptorships, new graduate programs, mentorship models, professional development and simulation-based learning. Interactive and learner-centred strategies catering for different learning preferences have been considered more effective than didactic, task-focused approaches to nursing education [13]. Preceptorships provide one-on-one support, which requires an experienced nurse being partnered with a new nurse in a formal relationship for a defined period [15]. Mentorship models are practices in nursing where an experienced nurse provides guidance, support and knowledge to a less experienced nurse [16]. Simulation learning replicates clinical scenarios in a safe and controlled environment, which allows learners to practice and refine clinical skills, critical thinking, communication and decision-making without risk to real patients [17]. These programs foster both emotional and professional support when transitioning to practice. In addition to structured clinical education programs, informal strategies, such as gratitude interventions, are reported to improve the wellbeing and resilience of graduate nurses during transition to practice [18].

These approaches, however, differ significantly in structure and are shaped by healthcare resources, educator expertise and workforce needs. There is a lack of clarity regarding the most effective structure for standardised education programs for NGNs across varying settings and the impact different approaches have on skill acquisition, confidence and retention. Current research focuses on specific approaches, such as nurse residency programs [7], mentoring programs [16] and simulation–based learning [14, 17].

Thus, there is a need to map the range of clinical education programs employed internationally to support NGNs as they transition into professional practice. A scoping review method was adopted [19] to provide a comprehensive overview of the reported clinical education programs, their outcomes and associated challenges. The findings will inform healthcare educators, policymakers and leaders in planning responsive strategies to strengthen new graduate clinical education programs and sustain a future-ready nursing workforce.

### Review question

What are the different clinical education programs and their impact on transition to practice for NGNs?

**Inclusion criteria**

- NGNs who have completed an undergraduate program leading to registration and are within 12 months of employment.
- Transition programs for NGNs in their first year of practice, including orientation, mentorship, preceptorship, simulation-based education, or digital/virtual learning.
- Outcomes related to job satisfaction, retention, clinical competence and self-confidence.
- Programs implemented in hospital or community settings (no geographical restrictions).
- Quantitative, qualitative and mixed-methods studies, literature reviews and quality improvement papers meeting inclusion criteria.
- Grey literature (e.g., theses, dissertations).
- Articles published from 2015 onward.

**Exclusion criteria**

- Programs involving nurses who have not completed a bachelor equivalent degree.
- Studies reporting only short courses or individual skill-building interventions.

## Methods

This study has utilised a scoping review method to explore current relevant data, inclusive of a diverse range of evidence. The JBI scoping review methodology as outlined in the JBI Manual for Evidence Synthesis was followed [19]. The review was registered and made publicly available in Open Science Framework https://osf.io/ke5hd, and did not deviate from the approach described in the published protocol.

### Search strategy

A three-step approach was adopted for the review. The initial search was conducted in CINAHL database and keywords contained in the title and abstract of relevant records were examined, along with the index terms used. A list of key words in the topic area was compiled, and a search strategy was performed by two reviewers (HE, JM) in consultation with an expert hospital librarian to ensure that relevant studies appeared in the search results. If any key concepts were missing, the search terms or filters were amended to capture relevant studies. In the second step, a search was conducted using the identified keywords and index terms in three databases; CINAHL, Medline and Scopus. The search was not limited to scholarly articles in each database, ensuring grey literature was identified, however a grey literature search outside of these databases was not conducted. The same search terms were used across the chosen databases (Appendix 1). This was followed by the third step of searching the reference lists of all included articles.

### Study selection

**Fig. 1:**
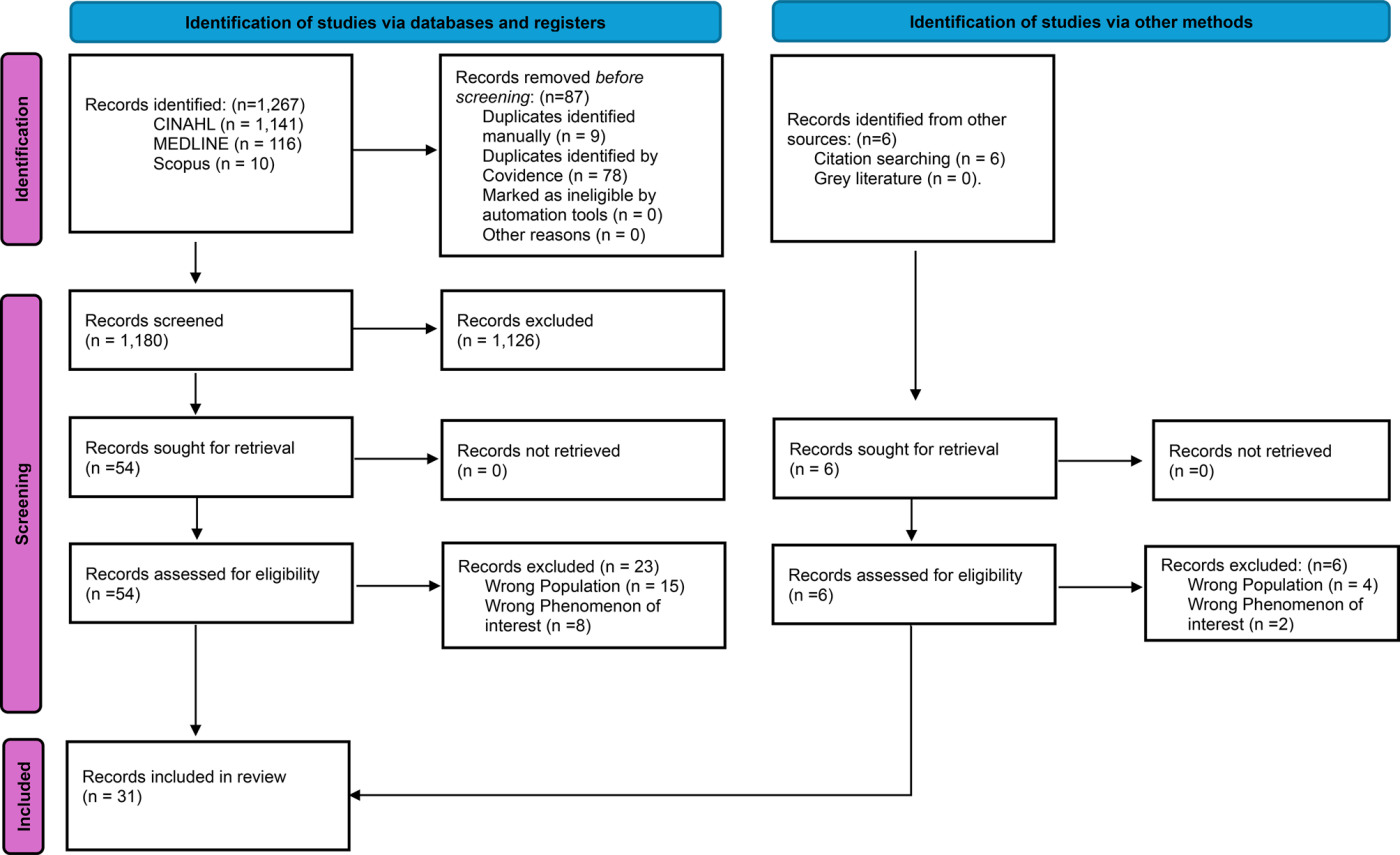
PRISMA flow diagram of the selection process

On completion of the search process, the identified records were uploaded to Covidence and duplicates were removed. The title and abstract of each article were screened against inclusion and exclusion criteria by two reviewers independently (CG, HE). Discrepancies were resolved by a third reviewer (JM or SPP). This was followed by a comprehensive full-text review of the selected articles along with the reference lists to determine the final selection of studies. Any conflicts for resolution were discussed as a group, and the search process is depicted using the PRISMA [20] in Fig. 1.

### Data extraction

The studies were distributed among five reviewers (NS, HE, CG, JM, SP), who independently extracted data using a structured Excel tool. The tool included: (1) Author, Year, Country (2) Phenomena of Interest (3) Methodology (4) Description of the clinical education programs and (5) Outcomes. Any disagreements that arose between the reviewers were resolved through discussion or with a third reviewer (SPP).

### Data analysis and presentation

The extracted data is presented in a comprehensive table (Table 1), within the results section. A narrative summary is presented to describe how the findings relate to the objective and review question for this scoping review.

**Table 1:** Characteristics of studies

Author, Year, country	Phenomena of interest	Methodology	Description of the clinical education approach	Outcomes
(Aggar et al., 2017) Australia	To assess if transition to professional practice program in general practice led to competent practice nurses in their first-year post-graduation.	Design: Longitudinal, exploratory mixed methods. Sample: 4 graduates and 7 preceptors. Mean age: Not given Data collection: Quantitative: Questionnaires at three time points (3,6,12 months) related to nursing competency, nursing experience and satisfaction Qualitative: Semi-structured interviews with participants after 12 months	The 12-month *Transition to Professional Practice* *in Primary Care Program* offering rotations through two practices for broader experience. Support included preceptors, educational resources, study days and networking opportunities, all overseen by a transition facilitator.	**Quantitative Results** -All graduate nurses were assessed as competent within their first year of clinical practice. -Both graduate nurses and preceptors reported high satisfaction with the program and the preceptor-preceptee relationship. **Qualitative Results** -Barriers: Limited understanding of the graduate nurse role in primary care, and variability in practice environments. -Enablers: Strong preceptor support, structured learning opportunities and a supportive practice culture. -Impact: Participants felt the program enhanced confidence, facilitated smoother transition and improved readiness for independent practice.
(Cadmus et al., 2023) USA	To assess experiences and outcomes in an out-of-hospital nurse residency program.	Design: Mixed methods. Sample size: 44 Mean age: 30.3 (14.6) Gender: Female 70.5% Clinical area: long-term care, assisted living, acute rehabilitation, home care, mental health and insurance companies Qualitative: Interviews Quantitative: Casey-Fink Graduate Nurse Experience Survey (CFGNES), and job satisfaction and modified preceptee program evaluation surveys.	*Out-of-Hospital Residency Program*: Bi-weekly educational sessions over 8 months, and development and completion of a quality improvement project. Program included mental health first aid training and had an experienced nurse as a preceptor and career counsellor. Training for preceptors was included.	**Quantitative Results** -100% reported that they would recommend the program. -90% identified that the residency helped them transition. -Residents increased in both confidence and competence. -Stress levels decreased by 2%. -All components of the CFGNES increased post residency, with the biggest increase in the patient safety category. **Qualitative Results** -Collaborated and built confidence in their role. -Had the opportunity to experience different specialties. -Expressed that they wanted to practice holistically.
(Cadmus et al., 2016) USA	Describe the development and implementation of a 12-month nurse residency program in long term care settings.	Design: Quality improvement, descriptive implementation design. Sample size: 37 new nurses, 37 preceptors. Mean age: 24.36 (SD 1.20) Data collection: Evaluation plan using 15 various measures, surveys and assessments at individual, unit and organisational levels.	*A 12-month nurse residency program*, including defined resource person, peer support opportunities and mentor. Education sessions, case studies, active learning and role-playing were part of the program.	- Retention rate was 86%. - Preceptors noted they had an increase in personal pride. - Nurse residents - overall very satisfied with the program and would recommend.
(Çamveren et al., 2022) Turkey	Examine the effect of preceptorship program on turnover intention, commitment and job satisfaction.	Design: Quasi-experimental (one-group pretest–post-test design). Sample size: 56 new nurses. Mean age 26.42 Female: 89.3% Data collection: 4 validated tools were used for demographic characteristics, turnover intention, organisational commitment and job satisfaction.	-*Preceptor training program* designed to enhance guidance skills, clarify role expectations, and equip preceptors with strategies for facilitating learning, providing feedback, and conducting evaluations. The validated scales measured: turnover intention; professional and organisational commitment, and job satisfaction.	-No significant difference in intention to leave unit, profession, organisational professional continuance commitment, professional normative commitment, or job satisfaction. -Intention to leave the organisation increased (t = -4.153, *p* < 0.001), decreases in organisational affective commitment (t = 4.443, *p* < 0.001), organisational normative commitment (t = 3.443, *p* < 0.001) and professional affective commitment (t = 7.390, *p* < 0.001).
(Cantrell et al., 2024) USA	To compare the effectiveness of a task-layered clinical orientation approach with traditional orientation methods in a registered nurse (RN) residency program.	Design: Non-randomised, comparative design. Sample size: 54 new RNs. Data collection: Surveys assessing clinical confidence, critical thinking and job satisfaction, including the CFGNES, new hire orientation-preceptor survey, and the Vizient/AACN Nurse Residency Program – Program Evaluation Survey.	The *task-layered clinical orientation* involved introducing new nurses to patient care tasks in a structured, layered manner, allowing for repetition and mastery of each task before progressing to the next.	-Average orientation duration: difference of 2.55 days. -Self-perceived competence (CFGNES): no significant differences between groups at any time point. -Preceptor evaluation (Vizient/AACN Survey): high satisfaction across both groups. -COVID-19: impact fewer post-COVID trainees “strongly agreed” that preceptors spent enough time with them (28% vs. 61% pre-COVID). -Orientation completion: no significant difference in completion rates between groups.
(Charette et al., 2023) Australia	To assess the effectiveness of transition programs on NGNs clinical competence, job satisfaction and perceptions of support.	Design: Longitudinal mixed-methods study. Sample size: 88 NGNs recruited during orientation. Data collection: Quantitative - Nurse Competence Scale (T0–T4), Nurse Satisfaction Scale (T4) Qualitative: Semi-structured interviews (T1–T4). At each time point, the questionnaire included demographic and the Nurse Competence Scale. At T4, also included the Nurse Satisfaction Scale.	*Transition program* emphasised gradual development of clinical competence. -Supportive environments that encouraged NGNs to ask questions and seek help. -Facilitated transition from student to professional nurse through preceptorship, structured orientation and education. -Two 6-month clinical practice rotations.	**Quantitative Results** -T1 (3 months): Significant increase in competence (+ 15.1 points); participants described “getting out of student mode.” -T2 (6 months): Felt more confident and in control. -T3 (9 months): No significant increase in competence. -T4 (12 months): Significant increase in competence helping role (*p* = 0.028), teaching-coaching (*p* = 0.005), ensuring quality (*p* = 0.046) and work role (*p* = 0.002). **Qualitative Results** -Participants reported high job satisfaction at 12 months. Satisfaction was closely linked to feeling supported and having access to help when needed. -A supportive environment encouraged confidence, autonomy and professional growth.
(Cline et al., 2017) USA	Evaluate the outcomes from an institutionally developed nurse residency program.	Design: Descriptive longitudinal evaluation. Sample size: 553 new RNs enrolled in the residency program. Data collection: CFGNES at the beginning and completion of the 12-month residency program. Retention rates at 1, 3 and 5-years post hire were measured.	*Institutionally developed nurse residency program* which included clinical orientation and 12 education days (one day per month). The study days included simulation with patient volunteers.	-Statistically significant change in scores in all domains (skills and procedure performance, work environment and role transition, job satisfaction, and comfort and confidence), except stress (*P* = 0.05). -Communication / leadership and patient safety scores increased in mean score from 2.88 to 3.24 (*P*<0.001). -The patient safety domain increased from 2.77 to 3.16 (*P*<0.001). -Mean scores in the support domain decreased from 3.36 to 3.29 (*P* = 0.002), and mean scores in the professional satisfaction domain decreased from 3.53 to 3.41 (*P*< 0.001). 1-year retention > 90%.
(Clipper & Cherry, 2015) USA	Evaluate the effectiveness of a structured preceptor development program.	Design: Project evaluation. Sample size: 59 graduate nurses: Age range: 21–25 yrs. Data collection: 16-item investigator-developed survey based on the attributes of transition shock theory. 7 demographic items; 8 questions (Likert scale) with 1 open-ended qualitative question.	*The Structured preceptor development program -* two groups of graduate nurses: those with trained preceptors vs. untrained. A blended learning format, with five online modules (approximately 3 h) completed in advance, followed by an intensive 8-hour in-person training session that delivered the core content.	-1-year retention rate at 89.5%, compared with 82.7% for the NGNs with untrained preceptors. -Mean of all survey questions higher among trained-preceptor groups except regarding intention to stay in current organisation > 12 months. -Significant findings for “develop collegial working relationships, positive work environment” and “adequate time to ensure a smooth transition”.
(Cusack et al., 2024) Australia	To explore the impact of transition program policy directives grounded in foundational elements of transition theory.	Design: Descriptive, mixed-methods study. Sample size: 158 NGNs and 8 nurse managers. Data collection: Quantitative: Online surveys at 1, 5 and 11 months using adapted Professional Role Transition Risk Assessment Instrument and the Professional and Graduate Capability Framework Qualitative: Telephone interviews with Nurse Managers at end of transition year.	*A structured transition program-*Five-day orientation program, mirrored shifts for four to five weeks, followed by the provision of an additional six months of preceptorship. Two six-month clinical rotations.	**Quantitative Results**: - Highest mean score across all surveys was in relationships (mean scores 4.5/ 5.0/ 5.2), indicating they felt accepted and comfortable approaching senior staff. -A strong majority (88%) indicated no intention to leave the profession. **Qualitative Results**: -Nurse Managers were generally able to implement transition program policies effectively. -The presence of a dedicated Graduate Nurse Coordinator with a manageable caseload was seen as critical to the program’s success.
(Forde-Johnston, 2017) United Kingdom	To develop, implement and evaluate a foundation preceptorship program for newly qualified nurses.	Design: Mixed methods. Sample size: 37 Data collection: Pre- and post-program evaluation forms, surveys, semi-structured interviews and focus groups.	*Foundation preceptorship program-*access to one-to-one support with the program lead, 16 study days, regular time with their preceptor and monthly clinical supervision using action learning sets.	-Improved confidence in practice with increased clinical supervision. -Improved preceptor-preceptee relationships. -Expressed a desire for regular reflection with experienced nurses and feedback from clinical experts to develop their clinical skills.
(Gellerstedt et al., 2019) Sweden	To describe NGNs experiences of the introduction process and leadership within a hospital trainee programme.	Design: Qualitative design. Sample size: 19 NGNs. Data collection: Focus group interviews. Qualitative content analysis: COREQ checklist used.	*A hospital-based trainee program* over eighteen months and includes rotation through four different clinics and four times per semester skills development, such as reflection, study visits and training days.	-Enhanced understanding of transition challenges. -Accessible and skilled managers were pivotal in aiding nurses’ development and confidence. -Themes: Need for an introduction when facing a complex reality; striving to stand on my own; the importance of having an accessible and multiskilled manager.
(Hardiman et al., 2022) Ireland	To explore the lived experiences of graduate nurses during the first six months of their new role within a person-centred graduate program.	Design: Qualitative. Sample size: 6 NGNs. Data collection: interviews -participative methods; nurses acted as co-researchers collecting and analysing their own and peers’ experiences.	*Person-centred graduate program* included 2-week induction and protected time to focus specifically on nurturing NGNs to feel confident, empowered and connected. Co-facilitators worked in partnership with work-based mentors. Inclusion of paid protected time for reflection and learning. Use of co-researcher model to empower nurses and enhance self-awareness.	-Felt safe to ask questions without judgement promoted confidence. -Paid protected time supported the transition from novice to confident staff nurse. -Being co-researchers provided the graduate nurses with awareness of the need to evaluate their practice and to use evidence to inform practice. -Caring and compassionate staff on the ward, and mentors promoted confidence.
(Henderson et al., 2015) Australia	To determine the value of the hospital graduate program to graduates’ assimilation and engagement in the workplace.	Design: Mixed methods. Sample size: 78 NGNs. Data collection: A survey approach based on valid tools, Clinical Learning Organisational Culture Survey. -Focus groups: Conducted with a subset of survey respondents.	*The Hospital Graduate Program*: Provided four days of paid hospital orientation then two weeks of supernumerary time, preceptor training, three study days.	**Quantitative Results**: - On average, experience was positive - Workplace components: affiliation, accomplishment, recognition and activate participation, rated highly. **Qualitative Results**: - Valued study days - Valued preceptors - Valued the nursing team - Valued being able to ask questions - The greatest challenge was after hours with reduce support.
(Hu et al., 2023) China	To investigate the effect of a transition program on ICU NGNs professional identity and their intention to remain employed.	Design: Quasi-experimental, one-arm pre‐ and post‐test design. Sample size: 53 NGNs. Data collection: Sociodemographic data collected at three time points. Professional Identity Scale for Nurses, adapted Intention to Quit Scale and Job Search Behaviour Scale.	*A structured transition program* with four 90-minute sessions of training, with one session per week. Received comprehensive guidance on teaching, learning, work and lifework balance from senior nursing preceptors. Six-month preceptorship and training for preceptors.	-Positive short-term effect on NGNs professional identity with total score increase from 111.5 to 114.2, *p* < 0.05 at one month but declined to 105.8 at the six-month follow-up. -No significant differences were observed in NGNs’ intention to remain employed across the three time points (*p* > 0.05).
(Hussein et al., 2016) Australia	To examine the influence of NGNs personal and situational factors on their satisfaction with the practice environment.	Design: Cross-sectional. Sample size: 109 NGNs. Data collection: The 26-item Manchester clinical supervision scale, Practice Environment Scale Australia and single-item 1–10-point Likert scale to assess the levels of confidence.	*Transition support program* 12-month program with two clinical rotations across a variety of clinical specialities. Includes orientation days and five education days.	- Younger nurses more satisfied with practice environment (*p* = 0.024) - NGNs in critical care areas were less satisfied (*p* = 0.037) - NGNs more satisfied with practice environment also more likely to be satisfied with unit orientation p = < 0.001) and more confident handling clinical situations (*p* = 0.043) - Independent and statistically significant predictors of satisfaction were: (1) unit satisfaction (standardised beta, b = 0.41); (2) satisfaction with the clinical supervision (b = 0.31); and (3) assigned unit: critical-care areas (b = − 0.17), explaining 32.5% of the variance.
(Hussein et al., 2019) [21] Australia	To examine the influence of NGNs personal and situational factors on their satisfaction with the practice environment.	Design: Post-test results of pre-post-test study Sample size: 87 NGNs. Data collection: Socio-demographic and situational data, Manchester Clinical Supervision Scale and the Practice Environment Scale Australia, investigator developed tool assessing intention to stay in clinical specialty.	*Transition support program* 12-month program with two clinical rotations across a variety of clinical specialities. Includes orientation days and five education days.	- Overall satisfaction with unit orientation 5–8 - Satisfaction with TSP 7–8 - Not having to practice beyond capability 2–5 - Intention to stay in unit directly related to placement in clinical situations beyond capability (*p* = 0.036) - Intention to stay in unit directly related to working in critical care (*p* = 0.003).
(Hussein et al., 2019) [22] Australia	To explore the clinical support experiences of NGNs and how these experiences influenced their learning, job satisfaction and skill development.	Design: Mixed methods; qualitative exploratory design. Sample size: 26 NGNs. Data collection: Baseline surveys and semi-structured interviews.	*Transition support program* 12-month program with two clinical rotations across a variety of clinical specialities. Includes orientation days and five education days.	-Identified themes included: - Clinical support facilitates learning - Conditions required for clinical support include support availability - Transforming me, role modelling
(Key & Wright, 2017) USA	Investigate whether implementation of the principles of cognitive apprenticeship during preceptorship of NGNs would impact their confidence and ease of transition.	Design: Mix-method Sample size: 51 NGNs Data collection: CFGNES	*Cognitive preceptor training and apprenticeship manual*, hospital skills checklist, unit orientation.	**Quantitative Results**: -4.3% increase in confidence from pre-test to post-test (pretest M = 3.00, post-test M = 3.18, *p* = 0.12). -10 items from the CFGNES showed a statistically significant improvement in confidence. − 37% identified improved orientation to feel more supported. **Qualitative Results**: -Confidence building was an ongoing process. -Experienced preceptors provided better experience.
(A. R. Kim et al., 2025) South Korea	Evaluate the effects of a critical reflection program utilising the Lasater Clinical Judgment Rubric (LCJR) reflective questions based on the Clinical Judgment Model (CJM) on NGNs clinical judgment skills.	Design: Non-randomised control trial. Sample: 153 NGNS. Data collection: Nurse Clinical Reasoning Competence Scale	*Critical reflective inquiry training program*: A six-week on-site training period, participants engaged in weekly one-on-one critical reflection sessions with their preceptors, guided by the structured reflective journal.	-Clinical reasoning competence significantly improved across both groups over time (experimental χ2 = 24.57, *p* < 0.001; control χ2 = 41.12, *p* < 0.001). - Clinical critical thinking skills showed no statistically significant change over time in both groups (*p* = 0.155, *p* = 0.099) - Clinical Judgement significant improved across both groups over time (experimental χ2 = 12.74, *p* = 0.002; control χ2 = 10.54, *p* = 0.005).
(H. Kim et al., 2025) South Korea	Evaluate the effect of a 10-week field-oriented training program for NGNs in critical care setting.	Design: Quasi-experimental (pre-post intervention). Sample size: 71 Data collection: Job Satisfaction Scale, Clinical Competencies, Professionalism Inventory tool, Organizational Commitment Questionnaire and short-term turnover rate.	*The 10-week field-oriented training program* including: Two-week modular orientation with classroom lectures, simulations, device practice and daily field practice. Eight-week individualised preceptorship offering one-on-one mentorship and preceptor training.	-Significantly improved clinical competencies (*p* < 0.001) and professionalism (*p* < 0.001). -Clinical knowledge (*p* < 0.001) and competencies (*p* < 0.001) significantly improved. - In contrast, job satisfaction (*p* = 0.004) and organisational commitment (*p* < 0.001) were significantly reduced over time.
(Y.H. Kim et al., 2018) South Korea	To evaluate the effectiveness of a work-based critical reflection program to enhance novice nurses’ clinical critical-thinking abilities, communication, competency and job performance.	Design: Quantitative-Quasi-experimental. Sample size: 44 NGNs. Data collection: Clinical Critical Thinking Skill test; Global Interpersonal Communication Competency Scale	*6-month critical introspection training program*, paired with reflective practitioners who had completed a 4-week preparatory course. Work-based guided critical reflection program using structured questions and feedback measuring: critical-thinking ability, communication competency and job performance.	**-**Clinical critical-thinking skills of those in the experimental group improved significantly (*p* = 0.003). -The differences in mean ranks of communication ability between two groups was significantly statistically different (*p* = 0.028). -Job performance improved significantly in both the experimental group and the control group, so there was no statistical difference (*p* = 0.294).
(Lap Fung Tsang, 2016) Hong Kong	To facilitate smooth psycho-social and professional reality integration for first-year preceptees during the transition to RNs.	Design: mixed methods. Sample size: 98 NGNs. Data collection: Clinical Competence Questionnaire, Occupational Stress Scale of Newly graduated Nurse, General Self-Efficacy Scale, in-depth interviews.	*12-month ToUCH program* through six stages. Educational methods included interactive and simulation teaching, demonstration, group case studies, activities sharing, presentation and reflection, round-up day camp. Clinical teacher to preceptee ratio 1:16.	**Quantitative Results**: -Significant improvement in occupational stress levels, ward management and interpersonal relationships (*p* < 0.0001) -Significant improvement in goal-setting, effort investment, persistence when encountering barriers and recovery from setbacks (*p* < 0.0001). -Resignations significantly improved from 11.8% to 3.9% **Qualitative Results**: - Felt more confident to handle different levels of clinical scenarios, increased sense of belonging, wanted to strengthen communication skills, counselling and support group were valuable.
(Oblea et al., 2019) USA	To evaluate the effectiveness of the Clinical Nurse Transition Program in improving readiness, confidence and competency among newly commissioned Army nurses.	Design: Prospective pre-test/post-test. Sample size: 86 newly commissioned Army nurses. Data collection: CFGNES and New Graduate RN Transition Program Competency Assessment Tool.	*26-week Clinical Nurse Transition Program*, including 936 h of supervised clinical practice, monthly didactics, and evidence-based practice projects; focussing on five core objectives including safe care, team leadership, decision-making, evidence use and professionalism.	-Significant improvements in confidence in managing patient loads, communication, delegation, and clinical decision-making (*p* < 0.0001). -Overall clinical competence and readiness to practice improved (*p* < 0.0001) -Participants reported the program effectively supported their transition into professional military nursing roles.
(Owings, 2016) USA	To evaluate the outcomes of a nurse residency program in a community hospital using the CFGNES.	Design: Secondary data analysis of a longitudinal survey. Sample size: 121 NGNs enrolled in the residency program. Data collection: CFGNES; retention and turnover data from hospital records.	*12-month community-based nurse residency program*, orientation, preceptor, monthly support sessions, evidence-based practice projects, and a structured curriculum. It aimed to strengthen clinical competence, critical thinking, clinical leadership skills and professional development during the transition to practice.	-Perception of social support declined (*p* > 0.05) -Ability to manage personal stress, no significant influence (*p* = 0.472) -Level of confidence with communication and leadership capacity significantly improved (*p* = 0.000) -Ability to organise and prioritise patient care significantly improved (*p* = 0.001) -Improvement in organising and prioritising patient care (*p* = 0.006) -Professional satisfaction no significant change (*p* = 0.075) -Level of comfort with technical skills improved -High retention (85%); turnover dropped from 37% to < 4%.
(Pelletier et al., 2019) USA	Effectiveness of Psychiatric–Mental Health Nurse Residency Program.	Design: Quantitative time-sequenced comparitive study. Sample size: 34 Data collection: On-line survey, 9 different validated tools and scales used, turnover data.	The *Psychiatric–Mental Health Nurse Residency Program* combining structured learning with peer support, formal teaching over 6 months with focus on quality, safety and enculturation.	-The study yielded a turnover rate of 11.7% in Year 1 (88.3% retention) and 2.9% in Year 2 (97.1% retention rate). - Improved scores in knowledge, skills, social support, coping self-efficacy, organisational commitment and reduced burnout over 24 months. - Most cited satisfier teamwork/coworkers (53%), disatisfier staffing levels/shifts (23%)
(Phillips et al., 2017) Australia	To explore how a continuous quality assurance feedback loop can support graduate nurse transition to practice and improve satisfaction during their first year.	Design: Mixed-method, four-phase study. Sample size: 34 NGNs Data collection: Online surveys, Likert-scale questions, open-ended responses, stakeholder workshops and interviews.	*Continuous quality assurance feedback loop* involving collecting monthly survey data on graduate nurse satisfaction across various domains with feedback to hospital management. Orientation, supernumerary periods, study days and performance reviews.	**Quantitative Results**: -No statistically significant difference in satisfaction between the two health services (*p* = 0.101). However, Health Service One which actively used the feedback reports consistently had higher satisfaction scores and showed improvement over time. **Qualitative Results**: - Enablers of successful transition: strong team and individual support, positive, welcoming culture, effective preceptor relationships, and time for debriefing and feedback. - Barriers: unrealistic expectations.
(Regan et al., 2017) Canada	To describe NGNs transition experiences in Canadian healthcare settings by exploring the perspectives of NGNs and nurse leaders in unit level roles.	Design: Descriptive-exploratory qualitative study. Sample size: 42 NGNs and 28 nurse leaders. Data collection: Focus groups and interviews; inductive content analysis.	The *transition to practice facilitators* were explored - themes around facilitating and impeding factors, challenges and intention to stay were identified.	-Orientation provided concrete information -Mentors at the point of care was described as important to build confidence. -Inadequate staffing impacted their ability to practice safely. -Lack of a consistent preceptor or mentors resulted in inconsistencies in learning. - Uncivil or intimidating behaviours impacted on their learning. -Staff often expected NGNs to perform beyond their readiness. - New Graduate Guarantee Initiative helps survival
(Rossler & Bennett, 2017) USA	Explore NGNs perceptions of the addition of human patient simulation into a med-surg residency program to promote transition into the clinical practice setting.	Design: Qualitative pilot study. Sample size: 15 NGNs. Data collection: Reflective journal and interview; thematic analysis.	*10-week simulation-based education integrated in a nurse residency program* with five simulation-based education sessions, a two-week orientation focused on hospital policies and procedures, didactic theory classes to reinforce nursing knowledge, and immersive competency-based skill stations to enhance clinical proficiency.	**-**Nurse Life (essential attributes for real work - teamwork, communication, be prepared); simulation exposure helped transfer knowledge and skills into practice. -Real Life practice (how to lead, how to speak up); simulation taught them how to speak up, lead patient care.
(Salmond et al., 2017) USA	Determine the effects of a residency program on new nurses’ confidence, competence, retention, job satisfaction, and perceptions of organisational safety.	Design: Pretest-post-test mixed methods. Sample size: 37 nurse residents, 39 preceptors. Data collection: Surveys including CFGNES and focus groups specifically designed program evaluation for both organisation level and individual preceptor and nurse resident outcomes.	The *nurse residency program* with classroom sessions, monthly learning collaboratives, and one-on-one preceptor support. Preceptors received 5 days of training, while nurse residents participated in 19–20 days of education plus 4 days of classroom and clinical site engagement.	**Quantitative Results** -Meaningful increase in “communication/leadership, patient safety, personal satisfaction” scales. -100% helped build confidence and skills. -86% retention rate. -Overall, 95% positive response on favourable effect on clinical practice. **Qualitative Results**: -Improved confidence and knowledge supported transition. -Enhanced patient care. -Increased commitment to long term care.
(Song et al., 2024) South Korea	Examine the effect of a nurse residency program on clinical competence, job satisfaction, organisational socialisation, turnover intention and turnover rates.	Design: Quasi experimental design with a non-equivalent control group post-test. Sample size: 167 NGNs Data collection: New nurse organizational socialization measurement tool, Minnesota satisfaction questionnaire, Clinical competence evaluation, Turnover intention measurement tool.	*Nurse residency program* including unit-specific simulation sessions at 2, 4 and 6 months, systematic education program, one-on-one small group training, individual counselling, advice and emotional support, stress management and team building activities, and 6 months of ongoing nursing support.	- Significantly higher clinical competence scores (M = 109.69) compared to the control group (M = 102.63), t = − 3.301, *p* = 0.001. -Higher job satisfaction (M = 60.09) than the control group (M = 57.27), though the difference was not statistically significant (t = − 2.227, *p* = 0.27). -Organisational socialisation: Scores were significantly higher in the experimental group (M = 125.94) versus the control group (M = 122.64), t = 1.783, *p* = 0.038. -Had a lower turnover intention score (M = 11.75) compared to the control group (M = 13.46), t = 2.696, *p* = 0.009. -Had a lower actual turnover rate (11.2%) than the control group (19.7%), though the difference was not statistically significant (χ² = 4.180, *p* = 0.42).
(Zhang et al., 2019) China	Assess the effectiveness of one-on-one mentorship program in reducing turnover rates.	Design: Longitudinal, non-randomised control study. Sample size: 438- 199 nurses (control group, 2013) vs. 239 nurses (intervention group, 2014). Data collection: human resource record analysis.	*One-on-one mentorship program* including 1 year relationship with mentor and career development plan. Preceptors given 4 h orientation program to develop mentoring skills, regular meetings to clarify mentoring objectives and responsibilities.	**-**Turnover rate was better with experimental group for the first year (*p* < 0.05) but not significantly different for 2nd and 3rd year (*p* > 0.05). -Total of 33% left from control group within 3 years; 14.64% in the experimental group.

## Results

The database search conducted in March 2025 yielded 1,267 records which were exported to Covidence. After removing duplicates, 1,186 records remained for title and abstract screening. Following title and abstracts screening, 1,132 irrelevant articles were excluded, resulting in 60 articles that underwent full text review. In total, 31 articles met the eligibility criteria for this review. The reasons for excluding 29 full-text papers are presented in Appendix 2.

### Characteristics of included studies

Geographically, 90% of the studies were from developed countries, the majority being from the United States of America (*n* = 11) [21, 23–32] and Australia (*n* = 8) [22, 33–39], with two studies from China [40, 41] and one from Turkey [42]. Within the 31 articles reviewed, there were 15 quantitative studies [21, 25–27, 29, 37, 38, 40–46], five qualitative studies [22, 31, 47–49], 10 mixed methods studies [23, 28, 32–36, 39, 50, 51] and a single quality improvement project report [24]. The majority of studies (74%) were conducted in a hospital setting, with three studies in specialty hospitals [26, 29, 30], three in long-term care facilities [23, 24, 32], one study specific to the critical care setting [40], and one assessed the orientation of NGNs in general practice [33]. Participant numbers across the studies varied substantially, ranging from as few as four participants [33] to as many as 553 [26]. Fewer than 50 participants were included in 45% of studies, while the remaining 55% involved sample sizes exceeding 50 participants. Larger sample sizes (> 50 participants) were predominantly associated with quantitative designs, with 13 out of 15 quantitative studies in this range. The qualitative studies all involved sample sizes of < 51 participants. Eight out of 10 mixed methods studies had sample sizes between 10 and 100 participants.

### Varied education programs

The 31 articles outlined an array of clinical education programs designed to transition the NGN to professional practice. A range of terminologies were used to describe the induction approach. The most common terminology was transition or residency program, used in 20 studies [21–26, 29–35, 37–40, 46, 49, 51], preceptorship in four studies [27, 28, 42, 50] and critical reflection program in two studies [43, 45]. The other studies described their intervention as a trainee program [47], clinical support program [36], graduate program [48], field-oriented training program [44] and mentorship program [41].

The duration of the interventions ranged from six weeks to 18 months. 18 programs were conducted over 12 months [21–26, 32–39, 41, 42, 50, 51], with the next most frequent duration being six months [29, 30, 45, 46, 48], followed by four programs that were less than six months in duration [25, 31, 43, 44].

Seven main clinical education approaches identified in the studies are included in this review. These have been collated in Table 2.

**Table 2:** Clinical education program components

Approach	Detail	No. of studies
Teaching approaches	Mandatory training [34] Clinical competency assessments [35] Training resources [33, 40] Skills training [21, 31, 44, 46] Online/digital-based education [33] Study days [21, 22, 24, 26, 33, 34, 36–39, 47, 50] Presentations, lectures [21, 33, 40, 42, 44] Classroom education sessions [21, 23, 29–32, 43] Simulation, role play, standardised patients [24, 26, 31, 44, 46, 47, 51] Case studies [29]	24
Support approaches (psychosocial or professional development)	Preceptors [21–25, 27, 28, 32–36, 42–46, 50] Preceptor training [23–25, 27, 28, 32, 33, 36, 42–45] Mentorship [41, 48, 49] Career counsellor [23, 41] Post-residency scholarship [23] Relationship and trust building [21, 46, 48, 49] Psychological counselling or organised collaborative support time [21, 24, 36, 40–42, 46, 50, 51] Networking and group discussion [21, 24, 32, 33, 36, 40, 42, 43, 49, 51]	23
Onboarding	Facility orientation [21, 22, 24, 26, 28, 34–39, 41, 44, 46, 49, 51]	16
Leadership involvement	Senior management oversight and support [23, 24, 32, 34–36, 42, 46, 50, 51] Facility coordinator [22, 30, 31, 33–36]	14
Program rules	Supernumerary time [22, 25, 31, 34–39, 44, 48] Shift rules [35, 36] Clinical rotations [22, 35, 37, 38, 47] Mirrored shifts [35, 36] Task-layered approach [25]	12
Reflection and feedback	Reflective practice [31, 34, 43, 45, 47, 50] Clinical appraisal [34, 39] Feedback loops [39, 43] Professional portfolio [34]	7
Continuous improvement	NGN included as co-researcher [48] Quality improvement project [21, 23, 24, 29, 32]	6

The most common approach involved organised instruction delivered either online, face-to-face, in a simulated learning environment or with printed resources. Eleven studies provided specific details of the education topics covered [23, 24, 26, 30, 32, 36, 40, 43, 46, 50, 51], and centred on communication [23, 24, 30, 32, 40, 50], teamwork and collaboration [23, 24, 26, 30, 32, 50], leadership [23, 24, 26, 32], stress management [30, 40, 46, 51], evidence-based practice [23, 24, 30, 32] and clinical knowledge and skills [24, 26, 36, 46, 50].

The second most common approach was psychosocial or professional development support, such as preceptors or networking opportunities. Preceptorship was the most frequently listed individual education approach to support NGNs. Specific training to upskill preceptors was included in 12 of these studies. The preceptor training topics included adult learning theory [23, 27], transition shock [23, 27, 42], delivering feedback [23, 24, 27, 28, 42] and organisational socialisation [23, 42].

The third approach, onboarding, consisted of a standard formal facility orientation that was not necessarily specific to NGNs [21, 22, 24, 26, 28, 34–39, 41, 44, 46, 49, 51]. Leadership involvement encompassed a range of strategies involving senior organisational team members, such as nurse leaders, to promote belonging and provide program oversight and administration. Program rules incorporated workplace integration strategies, such as specific shift patterns, clinical rotations or supernumerary shifts [22, 25, 31, 34–39, 44, 47, 48]. Reflection and structured feedback opportunities were embedded in the interventions outlined in seven articles [31, 34, 39, 43, 45, 47, 50], and continuous improvement participation, such as quality improvement projects, were embedded in six programs [21, 23, 24, 29, 32, 48].

### Impact of clinical education programs on transition outcomes

This review demonstrated the positive impact of clinical education programs on confidence, competence, job satisfaction and retention, in transitioning NGNs to the workforce. Outcomes of the clinical education programs were evaluated using a researcher-developed measurement tool and psychometrically validated instrument Casey-Fink Graduate Nurse Experience Survey the most frequently utilised [21, 23–26, 28, 29, 32, 33].

Nineteen articles reported on the positive impact of clinical education programs on NGNs’ confidence [21–26, 28, 29, 31–35, 43, 47–51]. Time in the role and clinical experience were key factors for building confidence. The support provided by mentors, preceptors and compassionate senior staff at the point of care also contributed to increased confidence [22, 35, 47–49].

Fourteen studies reported on the impact of the clinical education programs on NGNs’ competence [21–23, 25, 26, 29, 32–34, 43–46, 51]. Within these, seven studies provided details on what was included in the measurement of competence, reflecting combinations of knowledge, and technical and soft skills [21, 23, 25, 29, 32, 43, 44]. Eight studies reported a significant improvement in competence [21, 26, 29, 34, 44–46, 51], and four studies noted improvement, although did not demonstrate statistical significance [23, 32, 33, 43]. Only one study [33] reported measuring competence objectively via preceptor observation. Patient safety was the most commonly reported area of improved competence where all studies involved a 12-month residency program [21, 23, 26, 32]. Where the measurement of competence was assessed over a 12-month timeframe, the greatest impact was noted at three to six months of the intervention, rather than six to 12 months [21, 34]. Clinical support was identified as essential for increased competence [22, 34].

Seventeen studies reported on the impact of the education programs on NGN’s job satisfaction [21–24, 26, 28, 30, 32–36, 39, 42, 44, 46, 48]. Key factors in job satisfaction included understanding the impact of the nursing role, team acceptance and support, and was reported in eight of the seventeen studies [22–24, 28, 34, 35, 39, 48]. Four studies reported no change in job satisfaction [21, 36, 42, 46]. A decrease in job satisfaction was reported in five studies [26, 30, 32, 33, 44], with factors such as a lower perception of work being challenging, limited professional advancement opportunities, lack of downtime, elevated stress and low salary.

Fifteen studies reported on the impact of education programs on retaining NGNs in the workforce [21, 23, 24, 26, 27, 30, 32, 35, 38, 40–42, 44, 46, 51]. Five out of the fifteen studies reported on staff retention only [24, 26, 27, 30, 32], with all demonstrating improved retention rates (> 80%) following program participation. 80% of these articles were related to a transition or residency program between 10 weeks to one year duration, while three studies [17, 18, 23] examined preceptor programs, which enhanced the guidance skills of experienced staff and supported learning for novice nurses. Turnover of staff, or attrition, was reported in four articles [21, 41, 44, 46], highlighting staff turnover was significantly lower (< 12%) when NGNs participated in programs. In addition, intention to leave was lower among NGNs who participated in transition programs in five studies [23, 35, 38, 42]. One article [21] reported on both retention and turnover, noting the turnover rate was lower with compulsory participation in a residency or transition program and the retention rate increased from 81% to 97%.

### Challenges and enablers

Whilst the positive impact of structured transition programs is demonstrated, review findings highlighted the challenges encountered by organisations in implementing such programs. Organisational barriers to effective implementation of structured programs for NGNs were frequently cited, with ten studies identifying staffing-related constraints [22, 24, 28, 32, 34, 35, 39, 42, 49, 50], most prominently involving staff shortages and high staff turnover rates [22, 28, 42, 49], which compromised the ability to meet goals related to orientation time, coordinating preceptor support and appropriate patient allocation. Additionally, competing demands between maintaining clinical activities and releasing NGNs for scheduled educational activities posed safety challenges [24, 32, 50], often resulting in reduced access to learning opportunities, inconsistent program delivery or resentment from co-workers.

Studies reported the significant role of preceptors in shaping the transition experience of NGNs. The positive influence of preceptorship was highlighted in nine studies [27, 28, 32, 33, 35, 36, 39, 40, 50], where it was associated with enhanced confidence, clinical integration and professional development. Conversely, six studies [23, 24, 32, 35, 39, 49] identified challenges and negative aspects of preceptorship. These included limited preceptor engagement, lack of availability, insufficient preceptor experience, changes in assigned preceptors, difficulties aligning shift patterns and constraints in identifying suitably qualified senior staff to serve as preceptors - particularly within smaller units.

The positive impact of a supportive culture emerged in this review. It was important for NGNs to feel comfortable to express uncertainty, seek clarification [34, 36, 47, 48], and to know help was available if needed. The benefit of formal feedback was consistently identified, including allocation of dedicated time for NGNs to meet with preceptors or senior staff for feedback [21, 24, 36, 39, 41, 42, 50]. Peer support and networking opportunities were also consistently highlighted [22, 23, 30, 36, 47, 50, 51]. These interactions facilitated structured and informal sessions for debriefing and sharing clinical experiences. Such engagements contributed to the development of supportive relationships and increased emotional commitment.

## Discussion

This scoping review synthesised evidence from 31 studies across 10 countries, providing a comprehensive overview of clinical education programs that support NGNs transitioning to professional practice. The positive impact of structured clinical education programs on competence, confidence, job satisfaction and retention aligns with current research, however the optimal content, format and length of transition programs remain unclear and inconsistent [52]. NGNs view the start of their professional employment as an opportunity to gain clinical experience, develop as competent practitioners and progress their career goals [53], therefore an ideal educational approach that aligns with these goals will promote professional development and consistency to the workplace. Across the 31 studies analysed, education programs for NGNs varied in their terminology and detail, but all described a structured program that intended to build clinical skills and provide psychosocial support. The most frequently used descriptor, “residency program”, stemmed from recommendations in “The Future of Nursing: Leading Change, Advancing Health” report (2011), for a specific transition-to-practice program to improve nurse retention [54]. The positive impact of these structured programs is well recognised [55], and some form of a transition-to-practice program is mandated in many countries [9]. The length of programs varied widely, as identified in other studies, but the majority ranged between six and twelve months, and there is some evidence to suggest this is an optimal timeframe [56, 57].

This review categorised seven key education approaches that were included to support NGNs in their transition to professional practice. While some of these approaches were more popular than others, the combination of approaches and the lack of specific detail regarding structure, content and time, restricted the capacity to determine the most appropriate approach. Less frequently used approaches such as reflection, feedback and quality improvement may represent important missed opportunities to strengthen NGNs’ professional growth and overall transition into practice.

Two studies [27, 28] in the review analysed preceptorship almost exclusively as the intervention and provided training for the preceptors with a demonstrated positive impact on confidence and retention of NGNs. The significant impact of preceptorship on NGN competence [58] and retention [56] indicated the relationship between preceptor and preceptee is important for success. Preceptor training was repeatedly mentioned and focused on communication, teamwork and adult learning theories. This aligns with research that shows the importance of ongoing preceptor training and support [59].

The finding that confidence for NGNs is a product of time and experience is also supported in the literature [9, 55, 60], with the greatest improvement occurring between six to twelve months of employment [52, 61]. In the development of confidence, the importance of a supportive environment, including the supportive impact of preceptors and mentors, was a common finding in the review [52]. Evidence reports that a more complex program that included other education approaches in conjunction with preceptors was superior in increasing confidence rather than preceptorship alone [62]. A logical extension of this suggests that structured skill development opportunities, increasing in complexity over time would be beneficial.

Competency in nursing is the ability to deliver safe, quality patient care, whilst encapsulating the values, attitudes, skills, judgement and knowledge specific to the area of practice [63]. It is a dynamic rather than static process, requiring a commitment to continued growth and learning. The review identified an increase in NGN competence as a result of participation in a transition program, which aligns with reported evidence [9, 55, 64]. However, as pointed out by Tyndall et al. (2018), studies relied on self-reported competence rather than objectively measured patient outcomes [65]. As subjective assessment introduces bias, more research is needed using objective data to validate this outcome. Measuring competency over time prompts consideration of whether the improvements arise from the implemented education approaches or from normal maturation [64]. Foundational research highlights the cognitive processes and actions of novice nurses differ significantly from more experienced nurses [66], with the first six months of practice being a critical time for knowledge and skill development [67]. Therefore, supportive transitions that gradually increase clinical responsibility and autonomy in line with developing skill sets are important considerations when designing education programs.

Sustaining the workforce was a prominent theme in the review results. Positive outcomes in retention rates, reduced intention to leave and attrition were often reported when NGNs participated in residency or transition programs [6, 9, 68]. However, it must be noted that these residency or transition programs lacked consistency in program length and were thus unable to determine true impact on sustaining a workforce. Additionally, retention rates are often calculated at the end of the program [9], not allowing for long term impact in sustaining the workforce. Therefore, the need for longitudinal studies is required in this area.

A NGN’s decision to remain or resign from a role is mainly driven by their level of satisfaction [41]. Job satisfaction was a measurable outcome in this review, with most articles utilising validated tools such as the CFGNES survey. The review identified a mixed result on NGN job satisfaction when participating in a transition program, and thus no definite impact can be concluded, which concurs with other literature [69]. The review also identified that having a sense of role clarity [70], contributions to continuous improvement practices [71] and salary concerns [72] contribute both positively and negatively to job satisfaction. The review highlighted that job satisfaction is a complex matter, and it is not exclusively influenced by education programs but also how NGNs integrate into an evolving healthcare system.

The challenges for organisations to deliver structured clinical education programs was well evident in this review, with resource limitations, workload demands, inconsistency in program delivery and staff shortages adversely affecting the transition of NGNs. This tension between service delivery and academic preparation of NGNs has been a theme in the broader literature, where they report NGNs feeling overwhelmed by expectations and underprepared for the realities of clinical practice [70]. The difficulties associated with high turnover of experienced nursing staff were underscored in the review. Common problems identified included insufficient training, preceptor workload that could include dual patient care responsibilities and a lack of training or commitment of the precepting staff as previously identified [73]. The increasing use of digital or online education platforms can address some of these challenges, increasing education efficiency and accessibility in a cost-effective manner [74].

A supportive organisational culture is vital for reducing stress, emotional exhaustion and staff turnover, whereas unsupportive workplaces have a devastating impact on NGN job satisfaction, organisational commitment and mental health [75]. Preceptors are the most common approach to support NGNs [9], and there is strong evidence for formal preceptor training and intentional pairings that align learning styles and personality traits [76].

These review findings illustrate the importance of support as a key theme in generating positive outcomes for NGNs. In practice, support included organisational support, perceived via provision of a structured learning program that included senior management and nurse leader involvement. Secondly, support was exhibited in skill development through targeted learning opportunities and the involvement of preceptors. Thirdly, preceptors and mentors provided support through assisting social integration and team acceptance, along with peer networking opportunities with fellow NGNs. Conversely, absent or ineffective support frameworks have been shown to negatively impact competence and confidence [52], retention [77] and job satisfaction [75].

The impact of preceptors is influenced by their knowledge of adult learning theory and learning styles, and managers and preceptors should be empowered to proactively build these skills [78]. Regardless of formal training, preceptor experience remains a critical determinant of their effectiveness in the role [78]. Structured preceptor training in soft skills such as trust-building, advocacy and conflict resolution demonstrates organisational commitment and investment in human capital. Preceptors displaying an authentic leadership style that encourages information sharing and transparency have a positive impact on NGN transition outcomes [75], and this should be included in preceptor development programs. Feedback is an important expression of support that builds motivation to improve, facilitates integration into a new role and workplace, and promotes a continuous learning culture [46].

This review highlighted that whilst structured clinical education programs offer substantial benefits, their success is contingent upon organisational factors, preceptor engagement, and a culture of support and feedback. Addressing these challenges through strategic workforce planning, investment in leadership development and preceptor training is essential to ensure effectiveness of NGN transition programs. The use of digital learning platforms, accessible workspaces to promote peer support opportunities, as well as prioritising and promoting a culture of shared responsibility for learning could mitigate some of these challenges. A standardised program to transition NGNs into the workforce spanning at least six to 12 months is recommended and should include trained preceptors, involvement from senior staff, and specific education and networking support opportunities.

### Strength and limitations

A key strength of this scoping review is its comprehensive synthesis of evidence from 31 studies across 10 countries, providing valuable insights into structured clinical education programs that support NGNs during their transition to practice. The review mapped a wide range of program types and outcomes, offering a foundation for future research and policy development.

However, several limitations should be noted. Firstly, the review was restricted to studies reporting structured clinical education programs, which may have excluded short-term or informal strategies that also influence NGN transition. Quality appraisal of included studies was not undertaken, consistent with the scoping review methodology, meaning the overall quality of evidence cannot be guaranteed. Limiting the population to NGNs within one year of practice holding a bachelor’s degree may have excluded studies involving diploma or equivalent nurses. Furthermore, the search was conducted across three databases only and no grey literature search was conducted outside of what was identified within these three databases.

The predominance of studies from developed countries limits generalisability to low- and middle-income settings, where resource constraints and contextual challenges differ significantly. This gap may reflect either a lack of formal transition programs in these regions or a research and publishing bias toward Western countries. Furthermore, no studies focused specifically on rural or remote areas, which face unique workforce challenges in recruiting and retaining nurses.

## Conclusion

This scoping review has mapped a diverse range of clinical education programs designed to support NGNs as they transition into professional practice. The evidence highlights that structured clinical education approaches, such as residency models, preceptorships, mentorship, simulation-based learning and reflective practice, play a pivotal role in enhancing clinical competence, confidence, job satisfaction and retention. These programs are most effective when embedded within supportive organisational cultures that prioritise senior staff engagement, structured orientation, and protected time for learning and reflection. The demonstrated significant improvement in patient safety is an important finding for healthcare organisations to justify the cost of these programs as well as invest in further research. The lack of objective assessments of NGN clinical competence is an underexplored dimension of patient safety measurement. Further, a longitudinal approach to measuring these outcomes could further strengthen the benefits of these education programs for NGNs and inform more efficient and effective use of finite healthcare resources.

Despite the demonstrated benefits, challenges such as staffing constraints, inconsistent preceptor availability and competing clinical demands have been shown to hinder program implementation and sustainability. Healthcare organisations must design flexible and resilient approaches to address the needs of NGNs.

The findings of this review provide pertinent insights for educators and leaders to inform the design and delivery of responsive, evidence-based clinical education strategies. Strengthening these approaches is critical to engaging and retaining a resilient, competent and future-ready nursing workforce.

## Supplementary Information

Below is the link to the electronic supplementary material.

Supplementary Material 1

## Data Availability

All data generated or analysed during this study are included in this published article and its supplementary information files.

## Abbreviations

CFGNES: Casey Fink Graduate Nurse Experience Survey
NGN/s: New graduate nurse/s
RN/s: Registered nurse/s
