# Supplementary material for: Clinical education programs for new graduate nurses: a scoping review
Source: BMC Nurs. 2026 Mar 24;25:412. doi: 10.1186/s12912-026-04561-8 (PMC13137532; doi:10.1186/s12912-026-04561-8)
Supplement: Supplementary file 1 — Supplementary Material 1 [file 12912_2026_4561_MOESM1_ESM.docx]

**Appendix I: Search strategy**

***CINAHL (EBSCOhost) <March 2025 >***

| **Search Number** | **Description** | **Records retrieved** |
| --- | --- | --- |
| S1 | clinical competence OR job satisfaction OR self confidence OR (orientation or onboarding or training or mentoring or mentorship or education) OR perceptorship newly qualified nurse OR inservice training OR (simulation training or simulation education or simulation learning) OR (digital education or digital learning) | 1,097,924 |
| S2 | new graduate nurses OR (newly qualified nurses or newly graduated nurses or novice nurses or new nurses) OR (new graduate nurse or new nurse or novice nurse or beginner nurse or early career nurse) | 32,876 |
| S3 | program evaluation OR (programs or services or interventions) OR pilot program OR (evaluation or assessment) | 3,056,800 |
| S4 | ((S1 AND S2 AND S3) | 1141 |

***MEDLINE (Ovid ) <March 2025 >***

| **Search Number** | **Description** | **Records retrieved** |
| --- | --- | --- |
| 1 | orientation OR mentoring OR preceptorship OR "in-service training” OR simulation training" OR “digital-based learning” OR “job satisfaction” OR “retention” OR “self confidence” OR “Clinical competence" | 167361 |
| 2 | new graduate.mp. or newly qualified nurses | 30812 |
| 3 | Program Evaluation/ or "pilot projects OR program evaluation" | 228733 |
| 4 | 1 and 2 and 3 | 116 |

***Scopus <March 2025 >***

| **Search Number** | **Description** | **Records retrieved** |
| --- | --- | --- |
| 1 | (TITLE-ABS-KEY ( clinical competence ) OR TITLE-ABS-KEY ( job satisfaction ) OR TITLE-ABS-KEY ( self confidence ) OR TITLE-ABS-KEY ( orientation ) OR TITLE-ABS-KEY ( preceptor model ) OR TITLE-ABS-KEY ( mentoring ) OR TITLE-ABS-KEY ( inservice training ) OR TITLE-ABS-KEY ( simulation ) OR TITLE-ABS-KEY ( digital education ) | 6,825,708 |
| 2 | ( new graduate nurses ) OR TITLE-ABS-KEY ( newly qualified nurses ) OR TITLE-ABS-KEY ( novice nurse ) ) | 6,440 |
| 3 | ( TITLE-ABS-KEY ( program evaluation ) OR TITLE-ABS-KEY ( intervention study ) OR TITLE-ABS-KEY ( pilot studies ) ) | 2,345,554 |
| 4 | 1 and 2 and 3 | 10 |

**Appendix 2: Studies ineligible following full-text review**

***Ineligible partcipants***

1. Bell S, Gorsuch P, Beckett C, McComas A, Boss K, Rose K. An Evidence-Based Initiative to Reduce New Graduate Nurse Turnover: Implementation of a Mentorship Program. Worldviews on Evidence-Based Nursing. 2025;22(2).
2. Coyne D, Tuer A, McCulloh Nair J. Novice Nurse Support Group: A Pilot Study. Journal for Nurses in Professional Development. 2020;36(1):12-32.
3. Evans K. Improving the Perception of Support Through Mentoring of New to Practice Nurses. Journal of Continuing Education in Nursing. 2025;56(2):45-7.
4. Franklin AE. Effectiveness of Simulation Preparation on Novice Nurses' Competence and Self-Efficacy in a Multiple-Patient Simulation. Nursing Education Perspectives (National League for Nursing). 2015;36(5):324-5.
5. Geist NC, Browning M, Messick A. Development of a Critical Reflection Mentorship Program to Enhance Critical Thinking Among Novice Nurses. Journal of Continuing Education in Nursing. 2024;55(9):456-60.
6. Irwin KM, Saathoff A, Janz DA, Long C. Resiliency Program for New Graduate Nurses. Journal for Nurses in Professional Development. 2021;37(1):35-9.
7. Justus PD, Appel SJ. Simulation With Advanced Care Providers in a Nurse Residency Program. Journal for Nurses in Professional Development. 2018;34(4):180-4.
8. Kennedy J, Astroth KM, Woith WM, Novotny NL, Jenkins SH. New nurse graduates and rapidly changing clinical situations: the role of expert critical care nurse mentors. International Journal of Nursing Education Scholarship. 2021;18(1):1-11.
9. Lambert C, Wiencek C, Francis-Parr J. Effect of Simulation-Based Training on the Self-Confidence of New Nurses in the Care of Patients With Acute Deterioration and Activation of the Rapid Response Team. Journal of Continuing Education in Nursing. 2023;54(8):367-76.
10. Navarro SP, Nolasco MPB. Implementation of Escape Room Simulation Activity for Nurses Transitioning to Clinical Practice. Journal of the New York State Nurses Association. 2023;50(2):33-9.
11. Norris H, New K, Hinsberg F. Patient Deterioration Simulation Education and New Graduate Nurses' Self-Confidence and Competence: A Pilot Study. Journal for nurses in professional development. 2019;35(6):330-6.
12. Pillai S, Manister NN, Coppolo MT, Ducey MS, McManus-Penzero J. Evaluation of a Nurse Residency Program. Journal for Nurses in Professional Development. 2018;34(6):E23-E8.
13. Soltysik S. Evidence You Can Use: Implementing a transition to practice program in Rural NY: Improving retention and satisfaction of New Graduate Nurses during the first year of practice. New York Nurse. 2024;9(2):11-2.
14. Sutcliffe A. 'Effective preceptorship can help improve new professionals' confidence'. Nursing Times. 2023;119(11):15-.
15. Tomooka M, Matsumoto C, Maeda H. Effectiveness of a preceptors' social support program to aid novice nurses' error experience on preceptors' skill and novice nurses' perception of social support: A quasi‐experimental study. Japan Journal of Nursing Science. 2024;21(1):1-17.
16. Torres DA, Jeske L, Marzinski SJ, Oleson R, Hook ML. Best Fit Orientation: An Innovative Strategy to Onboard Newly Licensed Nurses. Journal for Nurses in Professional Development. 2022;38(6):350-9.
17. Wilson T, Weathers N, Forneris L. Evaluation of Outcomes From an Online Nurse Residency Program. The Journal of nursing administration. 2018;48(10):495-501.
18. Yao X, Cheng G, Shao J, Wang Y, Lin Y, Zhang C. Development and implementation of a standardized training program for newly graduated mental health nurses: Process and preliminary outcomes. Nurse Education Today. 2021;104:N.PAG-N.PAG.
19. Zhu Y, Zhang Y, Wu J, Ge X, Zhang Y. Effects of a theory-driven hybrid online-offline mentorship program on transition outcomes of newly graduated nurses in their first year: A randomized controlled trial. Nurse Education in Practice. 2025;84:N.PAG-N.PAG.

***Ineligible Phenomenon of interest***

1. Africa L, Trepanier S. The Role of the Nurse Leader in Reversing the New Graduate Nurse Intent to Leave. Nurse Leader. 2021;19(3):239-45.
2. Beddingham E, Simmons M. Developing and piloting a new role to enhance the clinical learning environment. Nursing Management - UK. 2016;23(5):18-24.
3. Foodani MN, Amouei K, Negarandeh R. Nursing residency program: A solution for overcoming transition challenges for newly graduated nurses. Nursing Practice Today. 2025;12(1):1-5.
4. Graf AC, Nattabi B, Jacob E, Twigg D. Experiences of Western Australian rural nursing graduates: A mixed method analysis. Journal of Clinical Nursing (John Wiley & Sons, Inc). 2021;30(23/24):3466-80.
5. Greer-Day S, Medland J, Watson L, Bojak S. Outdoor Adventure Program Builds Confidence and Competence to Help New Graduate RNs Become “Everyday” Leaders at the Point of Care. Journal for Nurses in Professional Development. 2015;31(1):40-6.
6. Jung D, Lee SH, Kang SJ, Kim J-H. Development and evaluation of a clinical simulation for new graduate nurses: A multi-site pilot study. Nurse education today. 2017;49(ned, 8511379):84-9.
7. Lalonde M, Hall LM. Preceptor characteristics and the socialization outcomes of new graduate nurses during a preceptorship programme. Nursing Open. 2017;4(1):24-3.
8. Larsen R, Ashley J, Ellens T, Frauendienst R, Jorgensen‐Royce K, Zelenak M. Development of a new graduate public health nurse residency program using the core competencies of public health nursing. Public Health Nursing. 2018;35(6):606-12.
9. Palermo K. Mentoring New Graduate Nurses to Decrease Turnover: A Pilot Project. Journal of Christian nursing : a quarterly publication of Nurses Christian Fellowship. 2024;41(1):E10-E5.
10. Ward-Smith P, Peacock A, Pilbeam S, Porter V. Retention Outcomes When a Structured Mentoring Program Is Provided as Part of New Graduate Orientation. Journal for nurses in professional development. 2023;39(4):E75-E80.
